# Targeting tumor-associated macrophages to synergize tumor immunotherapy

**DOI:** 10.1038/s41392-021-00484-9

**Published:** 2021-02-23

**Authors:** Xiaonan Xiang, Jianguo Wang, Di Lu, Xiao Xu

**Affiliations:** 1 Department of Hepatobiliary and Pancreatic Surgery, Hangzhou First People’s Hospital, Zhejiang University School of Medicine, Hangzhou, China; 2 NHC Key Laboratory of Combined Multi-organ Transplantation, Key Laboratory of the Diagnosis and Treatment of Organ Transplantation, CAMS, Hangzhou, China; 3 Department of Hepatobiliary and Pancreatic Surgery, the First Affiliated Hospital, Zhejiang University School of Medicine, Hangzhou, China

**Keywords:** Gastrointestinal cancer, Immunotherapy, Cancer microenvironment, Tumour immunology

## Abstract

The current treatment strategies in advanced malignancies remain limited. Notably, immunotherapies have raised hope for a successful control of these advanced diseases, but their therapeutic responses are suboptimal and vary considerably among individuals. Tumor-associated macrophages (TAMs) are a major component of the tumor microenvironment (TME) and are often correlated with poor prognosis and therapy resistance, including immunotherapies. Thus, a deeper understanding of the complex roles of TAMs in immunotherapy regulation could provide new insight into the TME. Furthermore, targeting of TAMs is an emerging field of interest due to the hope that these strategies will synergize with current immunotherapies. In this review, we summarize recent studies investigating the involvement of TAMs in immune checkpoint inhibition, tumor vaccines and adoptive cell transfer therapies, and discuss the therapeutic potential of targeting TAMs as an adjuvant therapy in tumor immunotherapies.

## Introduction

Given the association of malignancies with subverted immune surveilliance,^1^ immunotherapies provide options for advanced cancer patients, and multiple clinical trials are underway.^2^ Despite the impressive results achieved in several clinical trials,^3–6^ obstacles have been encountered with the current immune checkpoint inhibitor (ICI)-based immunotherapies.^7–9^ Suboptimal efficacy is among the major concerns because previous trials suggest that the response rate to ICI monotherapy is limited, and the responses vary significantly across multiple tumors and among individuals.^10–13^

Accumulating evidence has suggested that immune suppression in the tumor microenvironment (TME) represents a major barrier to maximizing the clinical potential of immunotherapies.^14^ The TME is complex with diverse populations of nontumor stromal cells that impact tumor immune evasion, response to immunotherapy, and patient survival.^15^ In addition to cytotoxic lymphocytes (CTLs) and natural killer cells (NKs) that are generally considered effective antitumor immune cells, the TME contains a range of other cell types that are involved in the crosstalk with anti-tumor immune cells, including cancer-associated fibroblasts (CAFs),^16^ endothelial cells (ECs),^17^ and tumor-associated macrophages (TAMs).^18^ CAFs can induce a robust stromal reaction characterized by fibrotic extracellular matrix (ECM) and make the TME convert to an immune-excluded type via the transforming growth factor-β (TGF-β) signaling pathway.^19–21^ The tumor-associated vasculature is another hallmark of advanced solid tumors.^22,23^ ECs of tumor vasculature can not only inhibit antitumor immunity by establishing a selective immune barrier via the vascular endothelial growth factor (VEGF)/prostaglandin E2 (PGE2)-FASL pathway,^24^ but can also exacerbate the hypoxia condition with low pH and cause high interstitial fluid pressure, which is unfavorable for the infiltration and activation of CTLs and NKs.^19,20,22^

Macrophages are involved in various processes in both homeostasis and disease.^25,26^ With effector functions such as phagocytosis, antigen presentation, and the plasticity to secrete a wide variety of signaling molecules, they serve as an efficient “firewall” in regulating homeostasis.^26–30^ They are also dynamic populations, and the resident macrophage pool can be rapidly expanded by infiltrating monocytes under pathological states such as tissue damage, inflammation and malignancy.^20,31–34^ Macrophages in the TME can be roughly induced into two contrasting groups: classically activated “M1” macrophages and alternatively activated “M2” macrophages.^32^ M2 and small populations of M1 cells, also known as tumor-associated macrophages (TAMs), have been generally thought to be involved in tumor initiation, progression, angiogenesis and metastasis.^35^ Most relevant for patients, a high TAM infiltration is often correlated with poor clinical outcomes in a wide variety of tumors and is believed to decrease responses to standard-of-care therapeutics, including radiotherapy, chemotherapy and targeted therapy.^27,36–44^ However, the “M1-M2” macrophage dichotomy is too simple to describe their complicated roles in the TME.^32^ Recent data acquired using unbiased large-scale techniques might help discriminate among macrophage subpopulations and have unraveled a previously unrecognized complexity in macrophage polarization, far beyond the old dogma of the binary “M1-M2” binary system.^45^ Furthermore, significant dynamic changes in macrophage subpopulations were observed during tumor development and were correlated with the efficacy of immunotherapy.^37,46–49^ These findings suggest a better understanding of heterogeneous TAMs and their roles in immunotherapy will be critical for developing effective immunotherapies.^50,51^

**Fig. 1:**
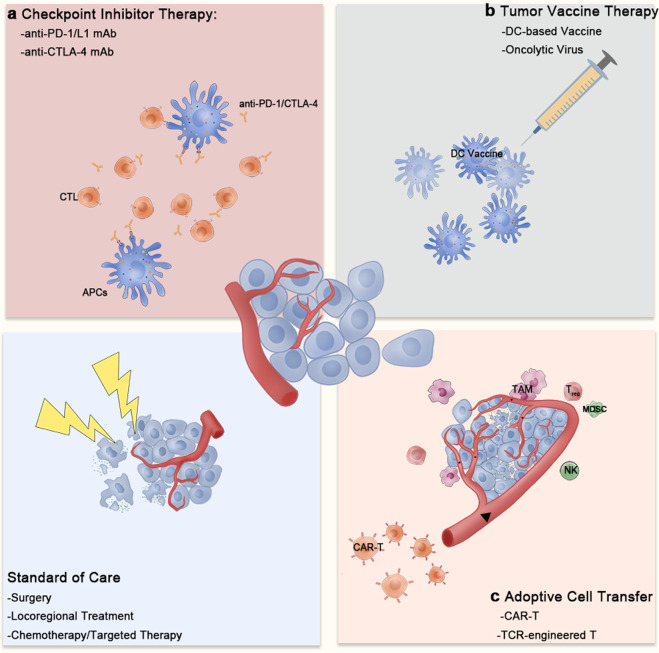
Classification of current tumor immunotherapies. The current tumor immunotherapies can be roughly divided into three types: **a** checkpoint inhibitors, including anti-PD-1/L1 and anti-CTLA-4 monoclonal antibodies; (**b**) tumor vaccines, including various dendritic cell-based vaccines and oncolytic virus-based vaccines; and (**c**) adoptive cell transfer, including CAR-T or TCR-engineered T cells. The antigens released from necrotic tumor cells during surgery, locoregional therapy, chemotherapy or targeted therapy enhance the immune recognition of tumor cells

Here, we attempt to illustrate the regulatory functions of TAMs in the TME and different immunotherapies. We further discuss the therapeutic potential of targeting TAMs to improve current immunotherapies (immunotherapy classifications are summarized in Fig. 1).

## TAMs in tumor initiation and progression

The strong relationship between inflammation and tumorigenesis has long been recognized.^52^ Approximately 90–95% of all types of tumors are connected to environmental exposures including tobacco, obesity, smoke, radiation, chemicals, and chronic infections, all of which could induce a smoldering inflammatory state.^53^ TAMs help establish a pro-inflammatory microenvironment and the link between TAMs and tumor initiation has been extensively studied in various clinical samples and preclinical models.^54–56^ For instance, liver macrophages were found to be the key source of steatosis-induced Wnt expression and the active Wnt/β-catenin signaling in macrophages can promote the growth of tumor progenitor cells, underlying the increased risk of hepatocellular carcinoma (HCC) and cholangiocarcinoma (CCA) in obese individuals.^57,58^

TAMs can promote tumor progression by producing mediators that remodel the tumor-supportive TME. Such mediators include growth factors and cytokines that support tumor cell proliferation; NF-κB-mediated factors that protect against apoptosis (for example, interleukin (IL)-1β, IL-6, tumor necrosis factor (TNF)-α, C-C motif chemokine (CCL)2, C-X-C motif chemokine (CXCL)8, and CXCL10);^35,51^ pro-angiogenic growth factors, such as VEGF, platelet derived growth factor (PDGF), TGF-β and fibroblast growth factor (FGF);^59–61^ and other factors that modulate tissue architecture and favor tumor cell migration, invasion and metastasis.^39,62–65^

TAMs also subvert local immune surveillance because they can directly reduce the activities of T cells and NKs by expressing cell surface proteins or by releasing soluble factors that display immunosuppressive functions (for example, arginase 1 (ARG1), indoleamine 2,3-dioxygenase (IDO), IL‑10, programmed death ligand 1 (PD-L1), and TGF-β)^65,66^ or indirectly suppress T cell activities through recruitment of other immune suppressive cells such as regulatory T cells (Tregs).^66,67^

Overall, TAMs play a dual role as “tumor promoters” and “immune suppressors” because they can promote tumor initiation and act as central drivers of the immunosuppressive TME through their expression of cell surface receptors, secreted cytokines, chemokines, and enzymes that regulate the recruitment and function of multiple immune cell subtypes.

## TAMs as regulators in immunotherapies

Numerous studies have shown the contribution of TAMs to immunotherapy resistance,^68^ while the precise mechanisms are still unclear. How these heterogeneous populations exert their distinct regulatory capability in response to different immunotherapies remains poorly defined.

### TAMs in checkpoint inhibitor therapy

**Fig. 2:**
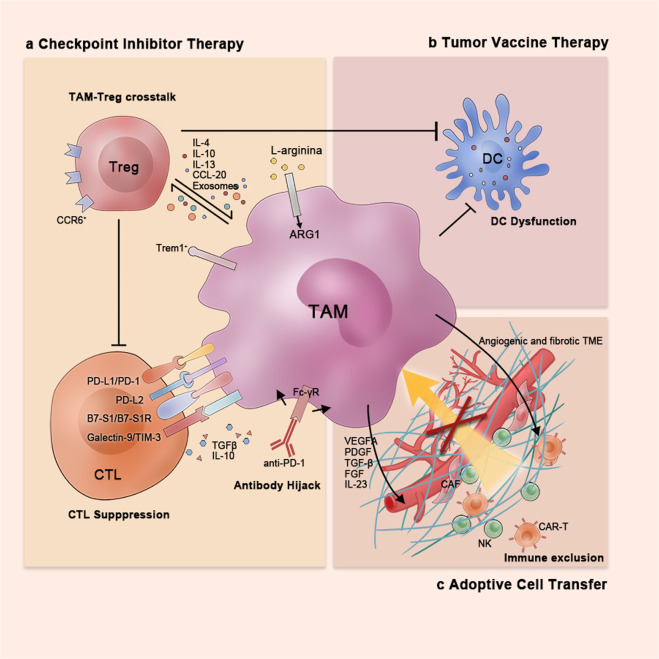
TAMs as regulators of tumor immunotherapies. TAMs exert their distinct regulatory functions in response to different immunotherapies: **a** In checkpoint inhibitor therapy, TAMs can suppress effective T cells directly via the expression of various checkpoint molecules and immunosuppressive cytokines and indirectly through crosstalk with Tregs and hijacking of anti-PD-1 antibodies. **b** In tumor vaccine therapy, TAMs can inhibit the antigen-presenting efficiency of dendritic cells; **c** In adoptive cell transfer therapy, TAMs prevent immune infiltration via build up the highly fibrotic and angiogenic TME

Inhibition of immune checkpoints, such as PD-1/L1 and CTLA-4, removes inhibitory signals of T cell activation, which enabling tumor-reactive T cells to overcome regulatory mechanisms and mount an effective antitumor response.^69,70^ However, the underlying cellular mechanisms remain unclear, namely, the expression patterns of checkpoint molecules and the interplay among ICIs and different components within the TME. In the setting of ICI therapy, the impact of TAMs should be carefully considered (Fig. 2, left).

#### Upregulation of checkpoint molecules

Early in the 2000s, the overexpression of checkpoint ligand B7-H1 (PD-L1) on tumor cells was considered a key mechanism of immune evasion.^71,72^ However, it was not until 2009 that two independent studies first demonstrated that macrophages are the predominant immune cells that express PD-L1 in HCC.^73,74^ These PD-L1^+^ TAMs, activated by tumor-derived IL-10, could mediate CD8^+^T cell dysfunction via the PD-1/PD-L1 interaction.^73,74^ Similar results were also observed in other tumor types including head and neck squamous cell carcinoma (HNSC), ovarian cancer, soft tissue sarcoma, bladder cancer and CCA.^75–80^ In the last decade, other B7 family checkpoints ligands were found to be expressed on TAMs, including B7-DC (PD-L2) and B7-H4 (B7-S1), as well as alternative checkpoints ligands such as galectin-9 and V-domain Ig-containing suppressor of T cell activation (VISTA).^81–86^ Thus, TAMs have been regarded as carriers of checkpoint ligands that are upregulated in response to TME-derived factors, resulting in immune exhaustion via the checkpoint ligand/receptor interaction in a cell-to-cell contact manner.

However, these findings do not provide a comprehensive picture. The expression of checkpoint molecules on macrophages might reflect the immune status within the TME. PD-L1 expression on macrophages, rather than on tumor cells, was positively correlated with patients’ overall survival (OS) and might be used as an independent prognostic factor based on a cohort study of 453 HCC patients.^87^ Surprisingly, PD-L1^+^ TAM-enriched tumors exhibited an activated immune status, with high levels of CD8^+^ T cell infiltration and immune-related gene expression, which indicates that a substantial proportion of tumor might be a amenable to ICI therapy.^87–89^

Moreover, the blocking effect of ICIs on the checkpoint molecules expressed on TAMs is increasingly attracting attention. Gordon et al. found that the phagocytic potency of PD-1^+^ macrophages is rescued by PD-1/PD-L1 inhibition, which lengthens the survival of colon cancer preclinical models in a macrophage-dependent manner.^90^ Another recent study based on a myeloid cell-specific PD-1 silencing model revealed the vital role of elevated intracellular cholesterol during anti-PD-1 treatment, which is required for differentiation of inflammatory macrophages and the promotion of antigen-presenting function.^91^ These findings show the complexity behind the checkpoint molecules expressed on TAMs. A further understanding of their intracellular regulatory mechanisms will be helpful for precise classification of TAMs and providing guidance for ICI treatment.

#### Crosstalk with regulatory T cells

Tregs are critical components of the TME and contribute to different aspects of tumor progression.^92^ Recent studies have revealed the compensation between TAMs and Tregs that derived immune evasion and ICI resistance.^93^

TAMs favor chemokine/cytokine-mediated recruitment of Tregs to the TME.^94–98^ TAM-derived CCL20 was found to promote the infiltration of CCR6^+^Tregs in colorectal cancer (CRC) and HCC, which may be an essential mechanism of anti-PD-L1 therapy resistance.^67,99^ Specifically, TREM-1^+^ TAMs elevate the expression of the chemokine CCL20 via the extracellular signal-regulated kinase (ERK)/NF-κβ pathway in response to hypoxia and tumor metabolites promoting infiltration of CCR6^+^ FOXP3^+^ Tregs.^67^ Thus, blocking the TAM-specific TREM-1 pathway could significantly reduce immunosuppressive Tregs recruitment, as well as restore the efficacy of anti-PD-L1 therapy.^67^ TAM-derived factors also play a central role in the induction of induced Tregs (iTregs) in the TME. It was shown that iTregs could be induced from CD4^+^CD25^−^ T cells co-cultured with M2-TAMs.^100^ A recent study by Zhou et al. highlighted the critical roles of TAM-derived exosomes in the induction of iTregs. They identified miRNAs enriched in exosomes, including miR-29a-3p and miR-21–5p which directly suppressed T cell-intrinsic STAT3 and regulated Treg/Th17 in ovarian cancer.^101^

Moreover, Tregs can further enhance the immunosuppressive properties of TAMs. In laryngeal squamous cell carcinoma (LSCC), malignant pleural effusion (MPE), and CRC, Tregs were found to promote the differentiation of monocytes into immunosuppressive TAMs directly.^100,102,103^ Tregs can also modulation of lipid metabolism in M2-like TAMs. Liu et al. found that Tregs could suppressed CD8^+^ T cell secretion of IFN-γ, which would otherwise block the activation of sterol regulatory element binding protein-1 (SREBP1)-mediated fatty acid synthesis in M2 TAMs. Thus, Tregs indirectly but selectively sustained M2-like TAM metabolic fitness, mitochondrial integrity and survival.^104^

Therefore, a positive feedback loop exists between TAMs and Tregs that further enhances their immunosuppressive effects in the TME.

#### Hijacking of anti-PD-1 antibodies

The constitutive expression of Fcγ receptor (FcγR) on monocytes/macrophages plays a crucial role in the antibody-dependent phagocytosis (ADCP) of tumor cells.^105^ However, it is worth noting that TAMs may have a significant impact on the pharmacokinetics and efficacy of ICI via Fc-FcγR binding. In mouse models and primary human immune cells, anti-PD-1 antibodies were observed to be seized by macrophages depending on the Fc domain of the antibody and the FcγR expressed by macrophages, which led to ICI therapy resistance.^106^ Moreover, a recent study has shown that Fc-FcγR binding-mediated TAM reprogramming can even induce hyperprogression in an non-small cell lung cancer (NSCLC) cohort and NSCLC patient-derived xenograft (PDX) models, although the mechanism remains unclear.^107^ Thus, how to interfere with the constitutive expression of FcγR on macrophages should be explored out in the tumor immunotherapy field. The effects mediated by the Fc domain of checkpoint molecular antibodies should be carefully evaluated and mechanistically understood.^108^

### Macrophages in adoptive cell transfusion

Adoptive cell transfer therapies such as chimeric antigen receptor T cell (CAR-T) or TCR-engineered T cell therapies enhanced the anti-tumor response in different advanced malignancies.^109–111^ The transferred cells must be trafficked and infiltrate into tumor sites to exert their cytolytic effects.^112^ However, this approach is not feasible for solid tumor treatment because of the relatively limited blood distribution and abnormal structure of tumor neo-vessels.^22^ In addition, tumors that develop from cirrhosis are highly fibrotic and difficult to penetrate physically.^16^ These features complicate the infiltration of adoptively transferred cells into tumor sites. In addition, TAMs contribute to the angiogenic and fibrotic TME (Fig. 2, lower right).

TAMs support tumor angiogenesis mainly by the production of factors such as VEGFA, PDGF, TGF-β and FGF.^59–61^ The subpopulation of TAMs characterized by the expression of angiopoietin 1 (TIE2^+^) in the blood or TME were considered to be close associated with intratumor neovessel formation.^113,114^ The molecular events of TAM-mediated angiogenesis were identified in a study based on a chronic HBV infection cohort, which showed that the individuals who finally developed HCC had higher serum levels of IL-23.^115^ IL-23, which is produced by inflammatory macrophages, enhanced macrophage-mediated angiogenesis by upregulating IL-23 receptor expression in macrophages. This “chronic inflammation-macrophage-IL-23” positive feedback loop might partially explain the significant role of macrophages in formation of the TME. Furthermore, high TAM infiltration correlated with a small number of IFN-γ-expressing active NKs in HCC,^116^ which might have a negative impact on the activation or survival of adoptive transferred NKs.

### Macrophages in tumor vaccines therapy

The identification of tumor antigens led to the development of tumor vaccination strategies in the 1980s. The host anti-tumor immune response is induced by tumor-antigen-pulsed dendritic cells (DC-based vaccines) or tumor-derived antigens released from lysed tumor cells (oncolytic virus).^117–119^ However, the results from clinical trials, were not as striking as expected.^120–123^ The limited efficacy of tumor vaccines in solid tumors may be ascribed to different possible causes, one of these being the strong immunosuppressive TME.^124^ As in other immunotherapies, studies have shown the accumulation of immune suppressive CD11b^+^ myeloid cells in response to the tumor vaccine treatment, which may result in therapy resistance^125–127^(Fig. 2, upper right).

Currently, TAM-targeting strategies combined with tumor vaccination are under evaluation. Anti-CD11b antibody-mediated depletion of myeloid cells showed a synergistic effect along with the vaccine by further prolonging the survival of tumor-bearing mice, although no significant reduction in tumor burden was observed.^128^ Injection of tumor lysate-pulsed DC also prolonged the survival of mouse models, and this therapeutic effect was further enhanced by injection of PLX3397, a CSF1R inhibitor that reprograms macrophages.^126^

## Therapeutic targeting of TAMs in tumor

**Fig. 3:**
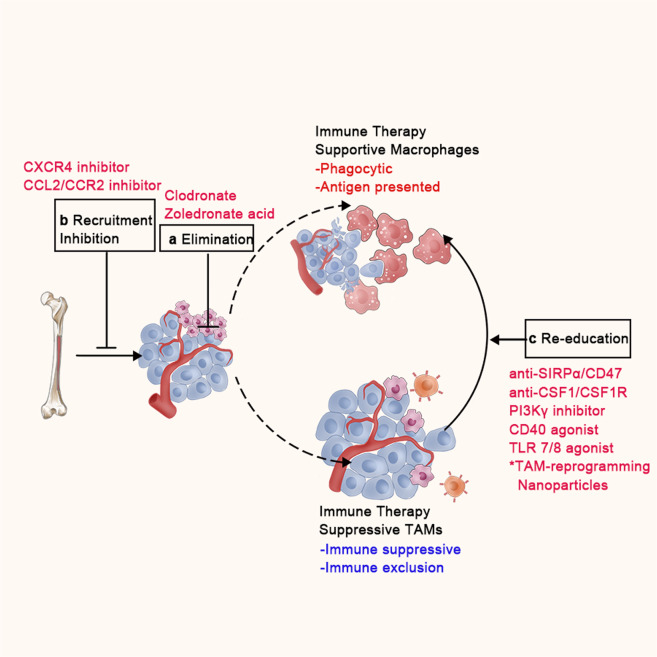
TAM-targeted Strategies in Tumors. TAM-targeted strategies can be roughly divided as follows: **a** elimination of macrophages already present in tumor tissue; (**b**) inhibition of monocyte/macrophage recruitment; (**c**) reeducation of TAMs toward an “immune-supportive” phenotype characterized by restored phagocytic and antigen presenting ability

Given that TAMs have a profound impact on tumor immunotherapies, there is considerable interest in the therapeutic targeting of TAMs to synergize current ICI-based immunotherapy. The different approaches that have been explored for targeting TAMs can be roughly sorted into three major categories: (1) eliminating TAMs already present in the TME; (2) inhibition of monocyte recruitment; and (3) reprogramming of TAMs (Fig. 3).^31,35,129^ These strategies have been investigated in preclinical models, and some have been translated into the clinical setting as adjuvant to immunotherapy.^130^ Here, we summarize current preclinical and clinical studies and discuss the potential strengths and weaknesses of these approaches in different solid tumors (Table 1).

**Table 1:** Combination TAM-targeted therapy with immunotherapy in selected clinical trials for tumor therapy

Mechanism of action	TAM-target	Compond	Clinical phase	Tumor type	Combinational immunotherapy	Results	ClinicalTrial
Elimination	Zoledronate acid	Zoledronate acid	Phase I/II (Completed)	Kidney Cancer and Lung Metastases	therapeutic autologous lymphocytes and IL-2	NA	NCT00588913
			Phase II (Terminated)	Metastatic Kidney Cancer	IL-2	NA	NCT00582790
Recruitment inhibition	CCR2/5 inhibitor	BMS-813160	Phase II (Ongoing)	NSCLC and HCC	Nivolumab (anti-PD-1 mAb)	NA	NCT04123379
			Phase I/II (Ongoing)	Locally advanced PDAC	Nivolumab (anti-PD-1 mAb) and GVAX (Tumor vaccine)	NA	NCT03767582
			Phase I/II (Ongoing)	Locally advanced PDAC	Nivolumab (anti-PD-1 mAb)	NA	NCT03496662
			Phase II (Ongoing)	Advanced RCC	Nivolumab (anti-PD-1 mAb)	NA	NCT02996110
	CXCR4 antagonist	BL-8040	Phase II (Ongoing)	Metastatic pancreatic adenocarcinoma	Pembrolizumab AND Chemotherapy	Tolerable and efficient	NCT02826486
			Phase I (Ongoing)	Metastatic, recurrent or stage IV PDAC	Pembrolizumab (anti-PD-1 mAb)	NA	NCT02907099
		AMD3100	Phase II (Ongoing)	HNSCC	Pembrolizumab (anti-PD-1 mAb)	NA	NCT04058145
Reprogramming	CSF-1R inhibitor	Pexidartinib	Phase I (Completed)	Advanced pancretic cancer or CRC	Durvalumab (anti-PD-L1 mAb)	NA	NCT02777710
		ARRY-382	Phase I/II (Completed)	Solid tumors, melanoma, NSCLC	Pembrolizumab (anti-PD1 mAb)	NA	NCT02880371
		BLZ945	Phase I/II (Ongoing)	Solid tumors	PDR001 (anti-PD1 mAb)	NA	NCT02829723
	Anti-CSF-1R mAb	IMC-CS4	Early Phase I (Ongoing)	Pancreatic cancer	Pembrolizumab (anti-PD-1 mAb) and GVAX (Tumor vaccine)	NA	NCT03153410
		Emactuzumab	Phase I (Ongoing)	Solid tumors	Atezolizumab (anti-PD-L1 mAb)	NA	NCT02323191
			Phase I (Completed)	Advanced solid tumors	RO7009789 (CD40 agonist)	NA	NCT02760797
		Cabiralizumab	Phase II (Ongoing)	Resectable biopsiable BTC	Nivolumab (anti-PD1 mAb)	NA	NCT03768531
			Phase II (Ongoing)	Advanced HCC	Nivolumab (anti-PD1 mAb)	NA	NCT04050462
			Phase I (Ongoing)	Solid tumors	Nivolumab (anti-PD1 mAb)	NA	NCT02526017
		AMG820	Phase I/II (Completed)	Advanced solid tumors	Pembrolizumab (anti-PD-1 mAb)	tolerable toxicity and moderate efficiency	NCT02713529
	Anti-CSF-1 mAb	MCS110	Phase I/II (Ongoing)	Solid tumors	PDR001 (anti-PD1 mAb)	NA	NCT02807844
		PD-0360324	Phase I (Ongoing)	Solid tumors	Avelumab (anti-PDL1 mAb)	NA	NCT02554812
	Anti-CD47 mAb	Hu5F9-G4	Phase I (Ongoing)	Solid tumors and ovarian cancer	Avelumab (anti-PD-L1 mAb)	NA	NCT03558139
			Phase I/II (Ongoing)	Urothelial Carcinoma	Atezolizumab (anti-PD-L1 mAb)	NA	NCT03869190
	SIRP-α Fc mAb	TTI-621	Phase I/II (Terminated)	Solid tumors	Diffent kinds of anti-PD-1/L1 mAb	NA	NCT02890368
			Phase I (Ongoing)	Hematologic Malignancies and solid tumors	Nivolumab (anti-PD-1 mAb)	NA	NCT02663518
	CD40 agonist	CP-870,893	Phase I (Completed)	Recurrent or stage IV melanoma	Tremelimumab (anti-CTLA-4 mAb)	NA	NCT01103635
		APX005M	Phase I (Ongoing)	NSCLC and metastatic melanoma	Nivolumab (anti-PD-1 mAb)	NA	NCT03123783
		Selicrelumab	Phase I (Completed)	Solid tumors	Atezolizumab (anti-PD-L1 mAb)	NA	NCT02304393
	TLR 7 agonist	DSP-0509	Phase I/II (Ongoing)	Neoplasms	Pembrolizumab (anti-PD-1 mAb)	NA	NCT03416335
		BNT411	Phase I/II (Ongoing)	Solid tumors and extensive SCLC	Atezolizumab (anti-PD-L1 mAb)	NA	NCT04101357
		LHC165	Phase I (Ongoing)	Solid tumors	PDR001 (anti-PD-1 mAb)	NA	NCT03301896
		Imiquimod	Phase I (Ongoing)	Solid tumors	Standard of Care PD-1 Therapy	NA	NCT04116320
	TLR 7/8 agonist	Resiquimod	Phase I (Completed)	Tumors	NY-ESO-1 Vaccination and Montanide ISA®-51 VG	NA	NCT00821652
		NKTR-262	Phase I/II (Ongoing)	Solid tumors	Nivolumab (anti-PD-1 mAb) and NKTR-214 (IL-2R biased agonist)	NA	NCT03435640
	PI3Kδ Inhibitor	Duvelisib	Phase I/II (Ongoing)	HNSCC	Pembrolizumab (anti-PD-1 mAb)	NA	NCT04193293
	PI3Kγ Inhibitor	IPI-549	Phase I (Ongoing)	Advanced Solid Tumors	Nivolumab (anti-PD-1 mAb)	NA	NCT02637531
	Pan-PI3K Inhibitor	SF1126	Phase I (Ongoing)	advanced HCC	Nivolumab (anti-PD-1 mAb)	NA	NCT03059147

### Macrophage elimination

The clearance of TAMs is an option to counter their negative impact directly during immunotherapy. Bisphosphonates, which are traditionally been used to prevent the bone metastases or excessive bone resorption, can be taken up by phagocytes and have cytotoxic effects on myeloid cells.^131,132^ Based on their structure, bisphosphonates can be divided into two categories: nonnitrogen-containing and nitrogen-containing bisphosphonates.^131^

Clodronate belongs to the family of non-nitrogen bisphosphonates. In early studies, clodronate-loaded liposomes (clodrolip) were often used to deplete liver macrophages.^133,134^ Liposomes are artificially prepared vesicles that undergo phagocytosis by macrophages after injection, and then, the intracellular release and accumulation of clodronate can induce apoptosis of macrophages.^135^ Administration of clodrolip depleted TAMs resulted in reduced tumor growth in preclinical models.^134,136^ The benefits of macrophage elimination have not only been seen with clodrolip, but also with other bisphosphonates, such as zoledronate.^132^ Zoledronate belongs to the third-generation nitrogen-containing bisphosphonate that has been shown to exhibit selective cytotoxicity towards matrix metalloproteinase-9 (MMP9)-expressing TAMs and to impair differentiation of monocytes into TAMs.^137^ Zoledronate acid (ZA) reduced the infiltration of TAMs, decreased tumor angiogenesis and inhibited tumor progression in different preclinical tumor models.^134,138–140^

A possible major barrier to this therapeutic approach might be the fact that general depletion of monocytes/macrophages is not TAM-specific and coincides with loss of tissue-resident macrophages that are crucial for maintaining homeostasis, and bacterial clearance, especially in the liver.^26^

### Macrophage recruitment inhibition

Another TAM-targeting strategy is to cut off their replenishment by circulating monocytes. The recruitment of circulating monocytes is highly dependent on several chemokine signals,^31^ and thus, interference with chemokine signaling using monoclonal antibodies or small molecule inhibitors might be an effective way to prevent TAM accumulation in the TME.

CCL2/CCR2 signaling plays a central regulatory role in circulatory monocytes and their infiltration into the TME, making it a promising TAM-targeted therapy.^141,142^ Inhibition of CCL2/CCR2 signaling has shown antitumor efficiency in different experimental animal models.^29^ Genetic silencing and administration of a CCL2 neutralizing antibody or CCR2 antagonist reduced the recruitment of circulatory monocytes, subsequently lowered the number of TAMs, and downregulated the secretion function of M2-like TAMs.^41,143–145^ More importantly, an enhancement of the function of tumor-infiltrating CD8^+^ T cells and NKs was observed during the treatment,^41,143,144^ which may suggest a good immunotherapy response. Thus, several phase I/II clinical trials are in progress to assess the therapeutic effect of BMS-813160, a small molecule inhibitor of CCR2/5, in combination with Nivolumab and/or the tumor vaccine GVAX in several solid tumors including HCC, NSCLC, renal cell carcinoma (RCC) and pancreatic ductal adenocarcinoma (PDAC).

Hypoxia-induced upregulation of stromal cell-derived factor 1 alpha (SDF-1α/CXCL12) also contributes to the recruitment of the suppressive M2 macrophages.^146^ Inhibition of the SDF-1α receptor (CXCR4) using the CXCR4 antagonist AMD3100 relieved regional immunosuppression and facilitated anti-PD-1 antibody treatment in a sorafenib-resistance HCC model.^147^ This study is of great translation value because hypoxia and HIF-1α activation are the most common and significant features of solid tumors and are usually aggravated during conventional treatments including chemotherapy, transcatheter arterial chemoembolization (TACE) and sorafenib treatment.^148^ Moreover, inhibition of CXCR4 might have synergistic effects with anti-angiogenesis drugs because TAMs can regulate the expression of CXCR4 via the ERK pathway, which is a novel vascular marker for angiogenesis.^149^ Therefore, CXCR4 antagonists, such as AMD3100 and BL-8040 should be judiciously considered in the future design of clinical trials for immunotherapies.

Although the efficacy of immunotherapies could be enhanced by myeloid cell recruitment inhibition, preclinical evidence from PDAC suggests that the resistance mechanism against this therapeutic approach may lie in the rapid compensation by tumor-associated neutrophils (TANs) and a lack of effect on tissue-resident TAM populations.^150,151^ Moreover, withdrawal of CCL2/CCR2 inhibitors may lead to a dramatically release of monocytes previously trapped within the bone marrow, which was shown to accelerate metastasis in a preclinical model of breast cancer.^152^ Although these limitations have not been reported in completed or ongoing clinical trials, considering them in the design of future clinical trials is critical, and alternative targets that overcome these limitations may be required for optimal and stable therapeutic responses.

### Macrophage reprogramming

An inevitable drawback to macrophage clearance and recruitment inhibition is the loss of their potential immune-stimulatory role as the major phagocytes and professional antigen-presenting cells (APCs) within the TME.^153^ Despite generally being tumor-supportive, TAMs may be phagocytic and suppress tumor growth by activating antitumor immune responses. This suggests that macrophage plasticity can be therapeutically exploited to restore the antitumor properties to TAMs.^25^ Thus, switching TAMs toward an “immune-supportive” phenotype provides an opportunity to reshape the immune-suppressive or exclusive TME and therefore presents a more effective approach to optimizing current ICI-based immunotherapies. This can be achieved by using therapeutics that promote macrophage polarization and/or using nanoparticles that can selectively reprogram macrophages to a restorative phenotype.^130^

#### Restoring phagocytic capacity

In homeostasis, normal cells can avoid self-elimination by phagocytes through the expression of anti-phagocytosis molecules,^154,155^ which are therefore called “phagocytosis checkpoints.” However, many studies have shown that tumor cells depend even more on phagocytosis checkpoints to evade immune surveilliance.^156^ Therefore, identification and intervention with phagocytosis checkpoints might provide a new approach for restoring the phagocytic capacity of TAMs to eliminate tumor cells.^157^

Signal regulatory protein alpha (SIRPα) is an ITIM-bearing inhibitory receptor expressed on myeloid cells, including macrophages.^157^ SIRPα recognizes CD47, which acts as a “don’t eat me” signal and is found to be overexpressed tumor cells and correlate with patients’ poor survival.^158,159^ Macrophage phagocytosis of tumor cells was restored after treatment with CD47 antibodies,^160^ and this macrophage-mediated phagocytosis was further enhanced in the presence of chemotherapeutic drugs, suggesting that patients with lower CD47 expression were more likely to benefit from adjuvant TACE treatment.^158^ It is worth noticing that CD47 is highly expressed in CCA.^161^ Interfering with the CD47-SIRPα interaction promotes phagocytosis in TAMs and consequently suppresses the progress of CCA.^161^ The unique overexpression of CD47 in CCA offers an exceptional opportunity for CD47-targeted therapy.

The bridging between innate and adaptive immune cells provides the rationale for combining phagocytosis checkpoint inhibitors with current ICI-based immunotherapies that boost the adaptive immune response.^157^ The potential for such combinations was initially observed when anti-CD47 therapy was shown to have synergistic effect with PD-L1 inhibitor in a mouse model bearing the B16F10 melanoma.^162^ Similarly, a bispecific antibody targeting PD-L1 on tumor cells and SIRPα on APCs showed a more significant antitumor effect against murine colon cancer compared with either anti-PD-L1 or anti-SIRPα monotherapy.^163^ Overall, these preclinical results along with earlier observations in ICIs confirm the notion that the conventional boundary between the innate immune checkpoint and adaptive immune checkpoints is becoming unclear, because more of these checkpoints have been found to function at both the innate and adaptive levels.^157^

#### Unleashing the immune-stimulatory capacity

The CSF1/CSF1R axis has been heavily investigated for its role in defining the survival, proliferation, differentiation and function of macrophages.^164–166^ Targeting CSF1/CSF1R signaling in protumoral TAMs represents an attractive strategy to eliminate CSF1R-dependent or reprogram M2-like TAMs.^167^ The altered TAM’s polarization will be key to reshaping the immunosuppressive TME and boosting a preexisting antitumor immune response.^167,168^ In preclinical models, CSF1/CSF1R blockade has been shown to improve the efficacy of different immunotherapies, including ICIs and adoptive cell transfer therapy.^169–171^ The positive results of these studies have led to clinical trials combining CSF1 and/or CSF1R inhibitors with ICIs or other immunotherapies.^172,173^

CD40, a receptor that belongs to the TNF receptor superfamily, is primarily expressed on APCs. The CD40-CD40L interaction upregulates the expression of MHC and promotes the secretion of pro-inflammatory cytokines, such as IL-12, which plays a significant role in T cell priming.^129^ Macrophage treatment with CD40 agonists in combination with anti-CSF1R antibodies resulted in profound TAM reprogramming before their depletion; these reprogrammed TAMs created a pro-inflammatory environment that elicited effective T cell responses, even in tumors that were nonresponsive to ICIs.^174,175^

Phosphatidylinositol 3-kinase γ (PI3Kγ) acts as a molecular switch that turns on an “immunosuppressive program” while shutting down “immune-stimulatory program”.^176^ Kaneda et al. showed that PI3Kγ determines the immunosuppressive properties of TAMs.^177^ It was shown that the lack of PI3Kγ activity in TAMs induced the expression of MHC-II and pro-inflammatory cytokines while reducing the immunosuppressive molecules including IL-10 and arginase.^177^ This dramatic shift of TAMs also enhanced adaptive immunity in the TME and significantly inhibited tumor progression.^177^ Another critical study by De Henau et al. also showed the potential of targeting myeloid-intrinsic PI3Kγ in overcoming ICI resistance.^178^ Further analysis is required to determine whether PI3Kγ inhibition could exert similar immunomodulatory function in other solid tumors.

The Lmdd-MPFG (LM) vaccine activates the NF-κB pathway in TAMs through the Toll-like receptor (TLR)2-MyD88 pathways, and recruits p62 to activate the autophagy pathway.^179,180^ The overall effect of LM skews the TAMs from the M2-like state into the M1-like state.^181^ Most importantly, this approach skewed the TME cytokine profile to anti-tumor profile, and this change restored the T cell reactivity to the anti-PD-1 blockade.^180^

#### Nanoparticles in the optimization of macrophages reprogramming

Systemic targeting of TAMs using nanomedicines is an attractive approach because TAMs are ideal therapeutic targets due to their considerable propensity to phagocytose nanoparticles.^182^ Notably, it has been reported that myeloid cells could take up ten-fold more nanoparticles than tumor cells in a preclinical model.^183^

Several recent studies have used nanoparticles loaded with TLR agonists or tumor peptides to promote reprogramming of the TAMs, exploiting the capacity of nanoparticles to both target TAMs and promote antitumor immunity. For example, the TLR7/TLR8 agonist R848 loaded nanoparticles preferentially accumulated in TAMs in mouse models and promoted macrophage reeducation.^184^ Another study based on immunotherapy resistance tumors showed that codelivery of a long peptide antigen, which induced antigen-presenting activity of TAMs, and TLR agonists to TAMs using a nanosized hydrogel (nanogel) can transform the resistant tumors into tumors sensitive to adaptive immune cell transfer.^185^

However, the development of TAM-reeducating therapies based on nanoparticles is still facing great challenges, such as how to preferentially deliver them to the protumoral M2-like TAMs or how to acquire a long-lasting and sufficient antitumor response.

Fortunately, engineering of new nanomedicines provides new opportunities by (1) applying nanoparticles modified with ligands that could recognize M2 TAM’s specific markers to achieve target delivery; and (2) preparing nanoparticles to reduce the number of TAMs in the tumor via specific cytotoxicity, or reeducating TAMs in a long-lasting manner with the carriers possessing drug controlled release properties.^186^ Effective development of such nanomedicines could lead to a breakthrough in the field of tumor immunotherapy.

## Conclusion and perspectives

TAMs are primary immune cells within the TME with high heterogeneity and complex roles as regulators of tumor immunity and immunotherapy. Thus, it is fundamental to reveal their exact regulatory mechanisms and identify macrophage-specific targets to optimize the efficacy of current immunotherapies. Recent studies have partially revealed the regulatory mechanisms and have highlighted three major TAM-targeting strategies: macrophage elimination, recruitment inhibition and reprogramming. Early clinical trials have focused on the first two approaches. Regardless, organ homeostasis disruption induced by resident macrophages and the potential metastasis-promoting withdrawal reaction remain key barriers to practical application in clinical settings. Going forward, a better strategy for macrophage reprogramming that attenuates their immune-suppressive ability while enhancing their potential immune-stimulatory functions is favorable for current ICI-based immunotherapy. However, the actual synergistic effect of macrophage-reprogramming agents, such as PI3Kγ inhibitors and CD40 agonists needs further evaluation. Moreover, macrophage reprogramming using nanoparticles has therapeutic potential in several preclinical models, but nanoparticle efficiency, safety and tolerability should be carefully evaluated in the human body.

Despite the recent progress in clinical and preclinical studies, some questions remain unanswered. For example, studies have highlighted the molecular events and signaling pathways of TAMs. Still, less is known about the intracellular metabolic switch during tumor progression and its potential impact on immunotherapy. Novel checkpoint receptors, such as T Cell Immunoglobulin and ITIM domain (TIGIT), VISTA or Lymphocyte-activation-gene-3 (LAG-3), have attracted broad interest, but what is the significance of macrophage populations expressing these different checkpoint receptors? Finally, because macrophages in the digestive system are direct sentinel cells for changes in the gut microbiota, understanding the exact mechanisms of these interactions and their consequences could potentially aid in tailoring an antitumor microbial cocktail. This concept is based on emerging studies suggesting that the manipulation of the gut microbiome can alter cancer incidence and the responses to immunotherapy.^180,187–189^
